# A VOICE-INTERACTION-MEDIATED SMART HOME TO SUPPORT AGING IN PLACE: A NEW PARADIGM TO ENGAGE WITH HEALTH-RELATED DATA

**DOI:** 10.1093/geroni/igz038.1659

**Published:** 2019-11-08

**Authors:** Yong K Choi, Hilaire J Thompson, George Demiris

**Affiliations:** 1 University of California Davis, Sacramento, California, United States; 2 University of Washington, Seattle, Washington, United States; 3 University of Pennsylvania, Philadelphia, Pennsylvania, United States

## Abstract

Current tools for health management such as personal health records (PHR), mobile applications, and wearable devices rely on conventional interfaces such as keyboard and mouse with screens for reading information. More recently, Internet-of-things (IoT) voice-activated smart speakers and embedded Artificial Intelligence (AI) assistants have emerged that may provide an effective user interaction platform to support healthy aging, creating a hands-free, conversational way to access and share health related data. We conducted an exploratory study with nineteen older adults (65+) who chose to evaluate a smart speaker for a 2-month as part of a larger IoT feasibility study. Three interview sessions were conducted to gather attitudes towards this technology. Participants provided feedback on future improvements or desirable features for health maintenance. A content analysis was performed to extract common themes. In general, participants expressed a positive experience with the voice interface and discussed its potential as an integral tool for their home health management. Based on these findings, we propose a voice interaction smart home platform to support healthy aging. This platform provides an intuitive voice user interface to execute commands to access, record, and query data from IoT sensors, a PHR and a hospital EHR. For example, the voice interaction platform will help individuals to dynamically explore their own health data thus eliminating manual and tedious searching of PHR data. We demonstrate how this platform can potentially improve usability issues including difficulties navigating charts and entering or retrieving health data using conventional interfaces. Finally, we highlight ethical and technical considerations.

